# Sol–Gel-Free Electrospinning of α‑Al_2_O_3_ Suspensions: A Systematic Study on Processing Commercial Powders into Ceramic Fibers

**DOI:** 10.1021/acsomega.5c11860

**Published:** 2026-06-02

**Authors:** Jesús Antonio Fuentes-García, Jonas Gurauskis

**Affiliations:** † Instituto de Nanociencia y Materiales de Aragón (INMA), CSIC-Universidad de Zaragoza, 50009 Zaragoza, Spain; ‡ Fundación Agencia Aragonesa para la Investigación y el Desarrollo (ARAID), Avenida de Ranillas 1D, 50018 Zaragoza, Spain

## Abstract

This study optimizes the fabrication of electrospun α-Al_2_O_3_ ceramic fibers from commercial powders, focusing on the synergy between solid loading (α-Al_2_O_3_ solid oxide), solvent systems (water/alcohol), and surfactant (PVP) molecular weight (K15 and K30) for suspension elaboration. Rheological characterization identified a highly suitable processing window with consistency indices (*K*) of 3.85–4.85 Pa·s and pseudoplastic flow (*n*) ranging from 0.927–0.972 for the elaborated suspensions. The dominant viscoelastic liquid character (*G*’> *G*”), high electrical conductivity (up to 1825 μS/cm), and reduced surface tension (58–64 mN/m) facilitated stable Taylor cone formation and enhanced jet thinning. A breakthrough was achieved by increasing solid loading and transitioning to high-molecular-weight PVP K-30. This strategy, coupled with isopropanol-based solvents, provided steric stabilization and structural recovery necessary to maintain single-fiber integrity during both spinning and sintering at 1500 °C. The resulting fibers exhibited high micrometric uniformity and morphological retention despite the formation of intermediate aluminum oxycarbides. This research demonstrates a robust, cost-effective, and scalable route for designing advanced ceramic scaffolds directly from industrial precursors.

## Graphical abstract

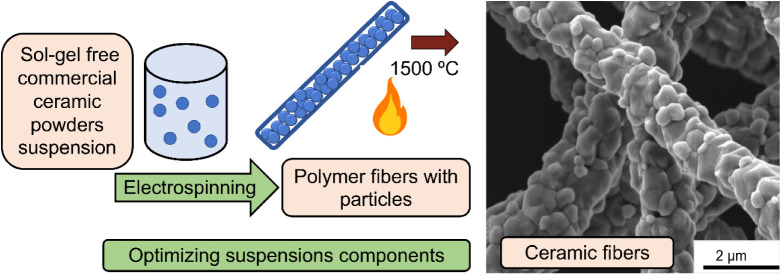

## 1 Introduction

Aluminum oxide powders in the alpha phase (α-Al_2_O_3_) are highly valued materials at an industrial level due to their high thermal stability, mechanical strength, chemical resistance, high surface area, porosity, and low cost.^1^ These robust and highly versatile properties position α-Al_2_O_3_ as essential for cutting-edge applications such as energy,^2–5^ aerospace,^6,7^ catalysis,^8,9^ filtration,^10–12^ among others.^13,14^

These applications can be significantly improved if the α-Al_2_O_3_ is processed into continuous filaments, particularly as ultrafine electrospun ceramic fibers (UECF).^15–17^ The exceptional properties of ceramic materials, combined with the unique advantages of fiber morphology (such as high surface area and interconnected porosity) for enhanced performance, make the controlled elaboration of α-Al_2_O_3_-based UECF (α-Al_2_O_3_–UECF) a relevant research task in materials science.^13,18,19^

However, the elaboration of controlled α-Al_2_O_3_–UECF has been a challenging task. Several strategies to produce α-Al_2_O_3_–UECF have been reported, primarily combining sol–gel reactions with electrospinning processing.^9^ This method relies on expensive metal–organic precursors (alkoxides), which are electrospun and subsequently calcined to form continuous ceramic fibers. While sol–gel methods offer precise control over chemical composition and homogeneity,^20,21^ they typically require expensive precursors and large volumes of organic solvents. This increases costs, extends processing time, and raises environmental concerns, ultimately impacting the practicality and efficiency of the method.^22^ These limitations render the sol–gel approach less suitable for industrial-scale applications where cost and time efficiency are critical.

In contrast to sol–gel-based electrospinning, which involves complex precursor decomposition and the release of hazardous residues based on chlorides and nitrates^23,24^ during the sintering process, the use of commercial α-Al_2_O_3_ suspensions preserves the crystalline phase identity and simplifies the thermal treatment profile. To overcome the challenges of the sol–gel route, the direct electrospinning of commercial α-Al_2_O_3_ powders in aqueous media represents a more straightforward and industrially scalable methodology. By dispersing readily available, less expensive commercial α-Al_2_O_3_ directly into a suitable polymer solution, the number of elaboration steps and timing are significantly reduced. This approach increases the reliability and scalability of α-Al_2_O_3_–UECF production.

Electrospinning is a versatile technique used to produce nanofibers from polymer solutions or suspensions by applying a high-voltage electric field. The working parameters, such as the working distance (WD), flow rate (FR), and electric potential (kV), play a key role in the formation and uniformity of the fibers.^25^ The stability and geometry of the Taylor cone, from which a charged jet of the polymeric suspension is ejected, are crucial for achieving continuous fiber morphology.^26^

However, the electrospinning of aqueous α-Al_2_O_3_ powder-based suspensions is still a major challenge. The main difficulty lies in creating a stable and spinnable fluid. Powders tend to aggregate, forming particles much larger than the diameter of the desired nanofiber. These aggregates can clog the nozzle or disrupt the continuous jet formation. The incorporation of solid particles dramatically affects the fluid’s behavior, leading to a complex and often unstable viscoelastic behavior influenced by colloidal interactions, surface tension, and electrical conductivity.^27–29^

Despite the clear benefits for industrial scalability, the systematic optimization of the electrospinning process for commercial α-Al_2_O_3_ powder suspensions to guarantee stable and continuous fiber formation remains an underreported area. The main novelty of this work lies in systematically establishing the optimal processing window for the direct electrospinning of α-Al_2_O_3_ powders using an aqueous soluble polymer binder. This study focuses on correlating the critical suspension properties (viscosity and electrical conductivity) with the electrospinning working parameters (WD, FR, and kV). The primary objective is to achieve stable jet ejection and controlled morphology, producing uniform and continuous α-Al_2_O_3_–UECF. The successful definition of this optimal processing strategy will pave the way for the cost-effective and large-scale production of high-performance α-Al_2_O_3_ ceramic preforms, directly impacting applications in advanced filtration and composite materials.

The scope of this work is focused on the systematic development and rheological optimization of a cost-effective, aqueous-based electrospinning process. By utilizing commercially available α-Al_2_O_3_ powders instead of expensive chemical precursors, this study establishes the processing parameters required to achieve morphological stability and single-fiber integrity, bridging the gap between laboratory synthesis and industrial-scale ceramic fiber production.

## 2 Results

The designed and presented multiparametric exploration elaborates on and evaluates a total of 12 α-Al_2_O_3_-based suspensions, all listed in Table 1. The sample labeling system maintains correspondence with the solvent employed during the ball milling for the α-Al_2_O_3_ paste elaboration: water (W) or isopropanol (ISO). The variation in the solids content was coded as an increase factor of the starting solid load, represented as 1X, 2X, and 4X. This increase in solids content was achieved by the controlled addition of α-Al_2_O_3_ powder to the base suspension (1X). The concentration of the utilized surfactant (K15 or K30) was consistently calculated at 1 wt % with respect to the total mass of the alumina powder incorporated.

**Table 1:** Suspensions Evaluated for the Electrospinning of Ceramic Fibers^a^

sample	surfactant	PVA (wt %)	PVA:α-Al_2_O_3_	FR (mL/h)	kV (kV)
W1X-8	K15	8	10:1	1.5	30
W2X-8	K15	8	10:1	1.5	30
W4X-8	K15	8	10:1	1.5	30
ISO1X-8	K15	8	10:1	1.5	30
ISO2X-8	K15	8	10:1	1.5	30
ISO4X-8	K15	8	10:1	1.5	30
ISO1X-10	K15	10	10:1	1	15
ISO2X-10	K15	10	10:1	1	15
ISO4X-10	K15	10	10:1	1	15
ISO1X^b^-10	K30	10	10:2	0.5	12
ISO2X^b^-10	K30	10	10:2	0.5	12
ISO4X^b^-10	K30	10	10:2	0.5	12

a The employed surfactant, PVA concentration, and PVA:α-Al_2_O_3_ ratio for the samples’ elaboration, and the parameters for the electrospinning flow rate (FR) and electric potential (kV) adjusted for a working distance of 15 cm are presented.

Since the final electrospinning suspensions were obtained by dispersing the postmilled paste in an aqueous PVA solution (at 8% or 10 wt %), the final number in the label refers to the employed PVA concentration. The PVA:α-Al_2_O_3_ ratio presented in Table 1 refers to the weight proportion of the final electrospinning mixture, specifically detailing the ratio between the PVA solution and the α-Al_2_O_3_ postmilled paste. The initial W and ISO series (samples W1X-8 to ISO4X-8 and ISO1X-10 to ISO4X-10) started with an initial solid loading of 15 wt % and used the K15 surfactant. While, the ISO1X^b^-10, ISO2X^b^-10, and ISO4X^b^-10 series also utilized ISO as the milling solvent, but they are distinguished by starting with an initial load adjusted to 32 wt % and the use of the K30 surfactant. Table 1 summarizes the employed surfactant, PVA concentration, and the PVA:α-Al_2_O_3_ weight ratio for each sample.

The WD during the electrospinning process of the suspensions was fixed at 15 cm in all cases. The FR was adjusted for Taylor’s cone formation, increasing the kV values as observed in Table 1. At higher solids contents, increased PVA concentration and superior surfactant molecular weight within the suspensions produced a reduction in the necessary inlet velocity for the fluids. Regarding the kV, it was also necessary to reduce the electric field for the stabilization of the produced Taylor’s cone.

For the observation of the effect of the different processing conditions on the fibers’ diameter and shape, typical SEM images were obtained. The diameter of the obtained fibers was evaluated using statistical analysis for average diameter determination after and before the sintering. The SEM analysis was crucial for determining the optimal parameters for both suspension elaboration and processing parameters, searching for controlled fiber formation during the electrospinning and its consolidation and fiber-shape retention after the polymer removal.

The morphology of the electrospun fibers using the W1X-8, W2X-8, W4X-8, ISO1X-8, ISO2X-8, and ISO4X-8 suspensions can be observed in Figure 1. Below each labeled image, an image of the same sample after sintering is presented. From the W1X-8 suspension, it was not possible to collect fibers under the employed electrospinning conditions. As obtained, the fibers from W2X-8 and W4X-8 showed uncontrolled fiber diameters and a network composed of poorly formed filaments. Promoted by the observed networking, the formation of islands of α-Al_2_O_3_ after sintering is observed.

**Figure 1:**
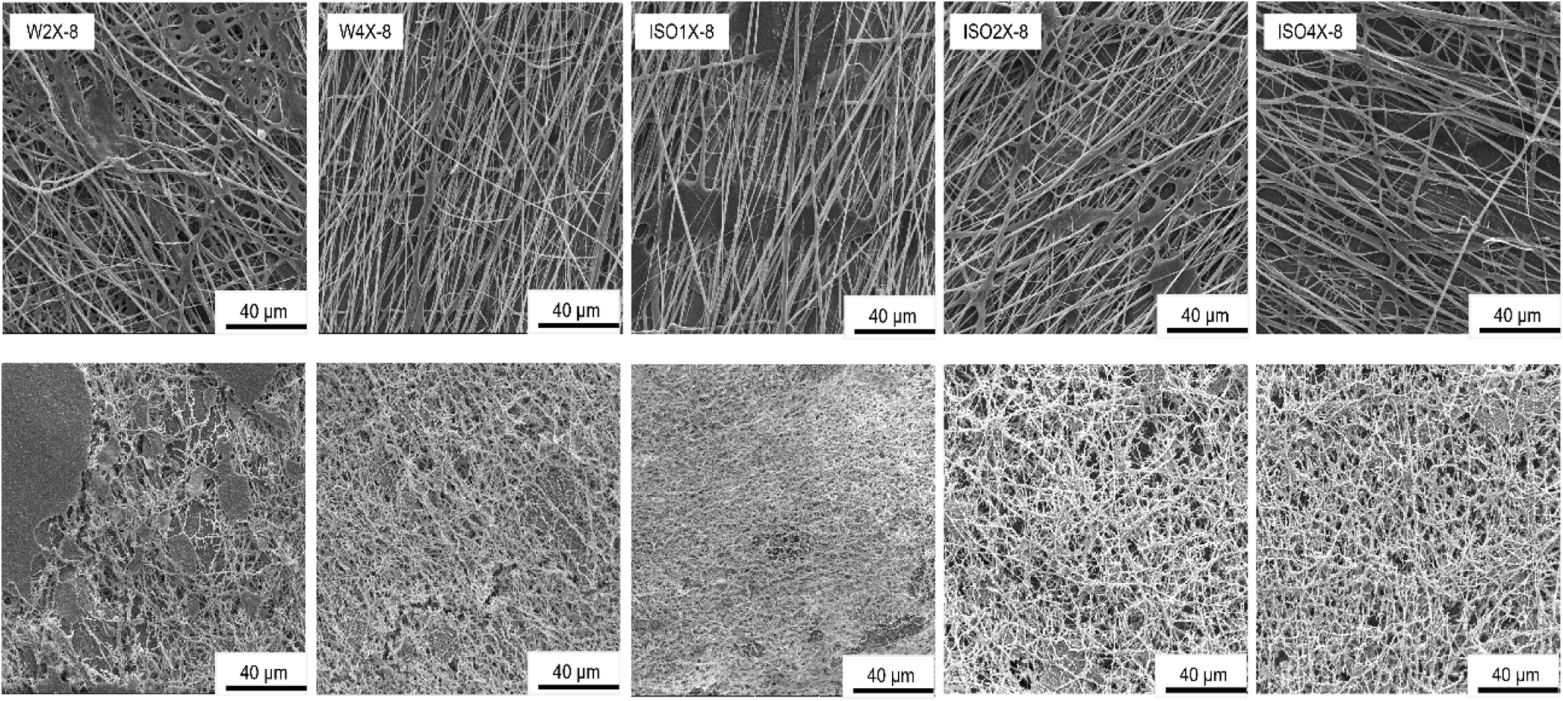
Effect of the solvent in ball-milling for electrospun fibers from aqueous PVA 8% binder. Electron microscopy images of W1X-8, W2X-8, W4X-8, ISO1X-8, ISO2X-8, and ISO4X-8 samples. In the upper panel, SEM images of the as-obtained fibers, and in the lower panel, the corresponding SEM images of the samples after the sintering process.

In the case of ISO1X-8, ISO2X-8, and ISO4X-8, even though the morphology is not controlled, the result of the sintering shows a fibrillar structure in a random orientation. However, fused fibers were observed, mainly in the parts where the fibers in the layered matrix form a union. It is important to highlight that the agglomeration of α-Al_2_O_3_ powders after the polymer melting is manifested mainly in two effects. A) The fusion of fibers, when the polymer melts, produces the conformation of the α-Al_2_O_3_ particles on a solid surface. B) Smaller particles result to be arranged in a fiber-like configuration composed of α-Al_2_O_3_ clusters. It suggests a polydisperse distribution of the α-Al_2_O_3_ powders within the suspension, influencing the final distribution of the particles forming a solid and nonporous surface in the case of the larger particles. In the case of the smaller particles, they can assemble into a fibrillar structure of clusters along the filaments.

The impact of the PVA’s concentration increasing (10 wt %) within the suspension on the electrospun fibers can be observed in Figure 2, where it is possible to observe thin, homogeneous, and continuous electrospun fibers, with a reduced formation of large clusters. According to the observed results, increasing polymer concentration has shown an improvement in the fibers’ morphology and electrospinning conditions, reducing the necessary electric field for their formation compared with the evaluated samples using 8 wt % PVA suspensions (Table 2). The obtained ceramic meshes from ISO1X-10, ISO2X-10, and ISO4X-10 samples after sintering can be observed in the SEM images of Figure 2.

**Figure 2:**
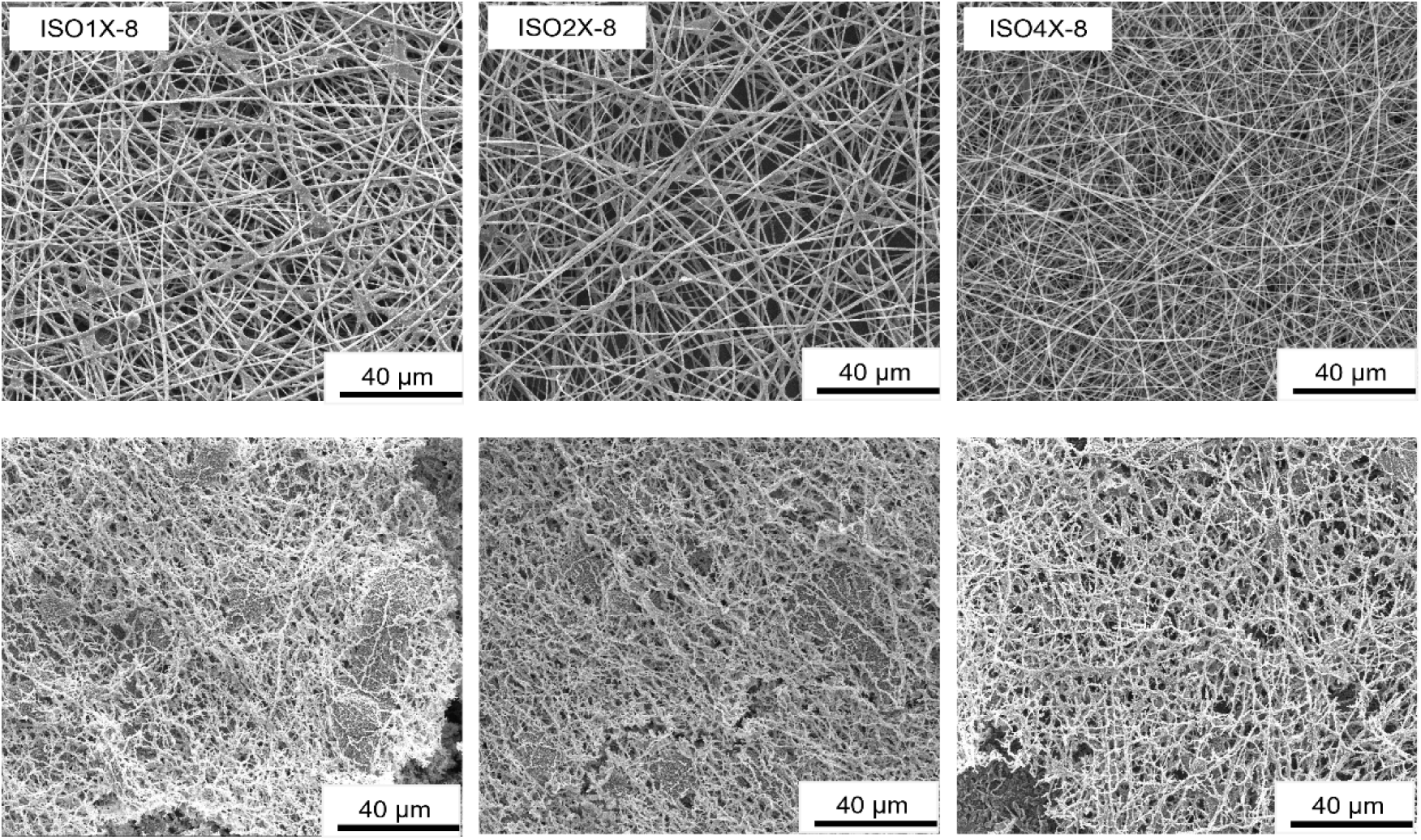
Effect of the binder concentration in α-Al_2_O_3_ electrospun fibers. Electron microscopy images of ISO1X-10, ISO2X-10, and ISO4X-10 samples. Well-defined electrospun fibers can be observed in the upper panel, and after sintering are shown in lower panel.

**Table 2:** Average Diameter (Ø) before and after Thermal Treatment (1500 °C)^a^

sample	Ø before treatment (μm)	*s* (%)	Ø after treatment (μm)	*s* (%)	Ø contraction (%)	macroscopic contraction (%)
W1X-8	X	X	X	X	X	X
W2X-8	1.14	49.12	2.03	27.58	FUSED	77
W4X-8	1.38	66.66	2.31	47.61	FUSED	72
ISO1X-8	1.23	43.90	X	X	X	76
ISO2X-8	1.06	68.86	0.89	46.06	16	75
ISO4X-8	1.59	49.68	1.06	43.39	33	70
ISO1X-10	1.00	55.00	0.79	35.44	21	72
ISO2X-10	0.90	41.11	0.97	46.39	FUSED	65
ISO4X-10	0.61	37.70	1.2	55.00	FUSED	63
ISO1X^b^-10	X	X	X	X	X	X
ISO2X^b^-10	2.60	25.00	1.50	42.00	20	35
ISO4X^b^-10	1.35	27.00	0.63	53.00	28	34

a Diameter statical analysis, contraction, and macroscopic contraction of the obtained α-Al_2_O_3_ fiber is presented. In case of X, it was not possible to determine the values.

Another strategy toward single fibers and avoiding the fibers fusing was to increase the α-Al_2_O_3_ solids loading. Suspensions labeled as ISO1X^b^-10, ISO2X^b^-10, and ISO4X^b^-10 (Table 1) were processed. The obtained α-Al_2_O_3_ fibers can be observed in Figure 3a and Figure 3b with EDS chemical characterization. Despite sample ISO1X^b^-10 being not possible to process, ISO2X^b^-10 and ISO4X^b^-10 samples showed improved formation and retaining of the fibrillar structure. The increase of the α-Al_2_O_3_ loading and the molecular weight of the surfactant were key factors in the formation of the ceramic fibers. The formation of fibers with larger diameters was observed for ISO2X^b^-10 compared with ISO4X^b^-10 samples. According to the images, it is possible to establish that the formed ceramic fibers are composed of the consolidation of α-Al_2_O_3_ particles with different sizes.

**Figure 3:**
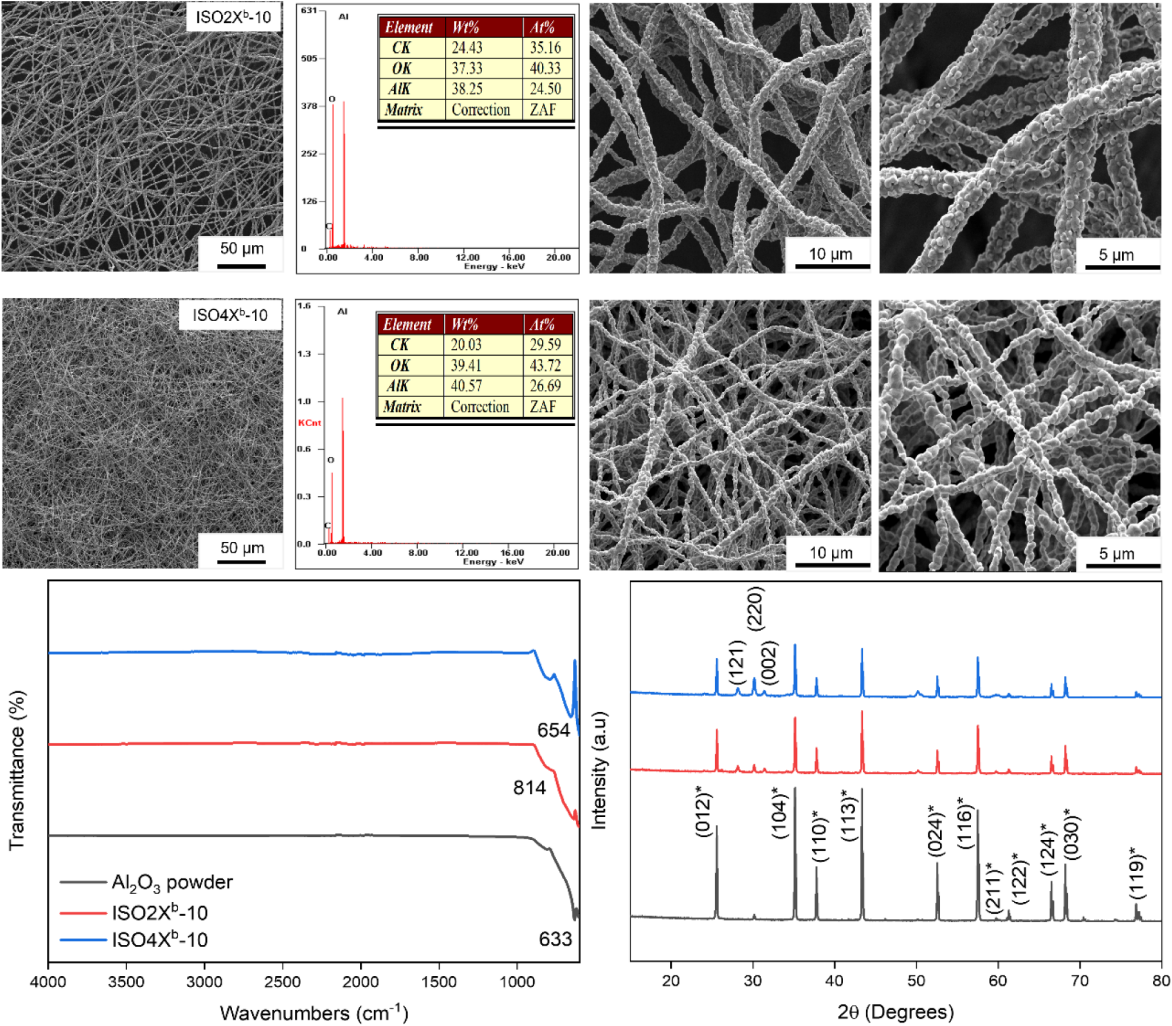
Effects of PVP K-30 and solids loading on electrospun α-Al_2_O_3_ fibers. Electron microscopy images at different magnifications of ISO2X^b^-10 and ISO4X^b^-10 samples are presented after the sintering.

The diameter modification of the produced fibers under the different electrospinning processing conditions for each elaborated suspension is summarized in Table 2. The average diameter and standard deviation (μm) before and after thermal treatment are presented. From the difference in these values, Ø contraction (%) was calculated. The increase of the diameter after the thermal treatment corresponds to fused fibers during the thermal treatment (FUSED). While macroscopic contraction was obtained by measuring the mat after and before the thermal treatment.

The chemical and structural evolution of the fibers was evaluated by comparing the commercial α-Al_2_O_3_ powder with the calcined ISO2X^b^-10 and ISO4X^b^-10. The results are presented in Figure 3c. The FTIR spectrum of the raw alumina powder is characterized by a sharp and well-defined absorption band centered at 633 cm^–1^, which is attributed to the characteristic Al–O stretching vibrations in the octahedral sites of the corundum structure. In contrast, the 2X and 4X fiber samples show a significant shift and the emergence of new signals. Intense bands at 654 cm^–1^ and 814 cm^–1^ are observed.

The presence of the 814 cm^–1^ band is particularly noteworthy, as it suggests the formation of different aluminum–oxygen–carbon environments. This frequency is often associated with Al–O vibrations in more distorted or complex lattice structures, such as those found in aluminum oxycarbides (Al_5_O_6_C_2_ and Al_4_O_4_C), where the presence of interstitial carbon modifies the standard metal-oxide vibration modes. This assumption is supported by the EDS results, showing carbon in the samples after the sintering process (1500 °C).

The XRD pattern of the starting powder (Figure 3 d) confirms a pure α-Al_2_O_3_ phase (corundum), with all characteristic reflections present between 15° and 80° (2θ). However, the ISO2X^b^-10 and ISO4X^b^-10 samples exhibit a distinct diffraction profile. While the primary α-Al_2_O_3_ peaks remain, three new prominent reflections appear at 28°, 30°, and 31°.

The reflection at 28° is highly characteristic of the (004) plane of Al_5_O_6_C_2_ (JCPDS 47–1522). The peaks at 30° and 31° correspond to the (102) and (103) reflections of Al_4_O_4_C (JCPDS 47–1521). The emergence of these phases confirms that, during the high-temperature treatment at 1500 °C, the residual carbon from the PVA/surfactant decomposition did not fully oxidize. Instead, it reacted with the α-Al_2_O_3_ matrix to form these intermediate oxycarbide compounds. The intensity of these peaks in the 4X sample suggests that higher solids loading (and consequently higher surfactant content) provides more carbonaceous precursors, promoting the stabilization of these carbide-related structures.

On the other hand, the properties of W1X^b^-10, W2X^b^-10, W4X^b^-10, ISO1X^b^-10, ISO2X^b^-10, and ISO4X^b^-10 suspensions were further characterized because of the successful formation of single fibers. The presented flow curves from the suspensions consistently exhibit shear-thinning (pseudoplastic) behavior across all solid loadings (Figure 4), confirming that the internal structure (PVA chains and/or alumina clusters) aligns under increasing shear stress.^30^ Regarding solid loading, the samples show a nonmonotonic trend, suggesting steric stabilization where the PVA binder effectively disperses the alumina particles, thus minimizing internal friction and hydrodynamic resistance.^31^ The 4X loading is the most viscous in both solvents (W or ISO), dominated by the high volume fraction of solids and the resulting severe hydrodynamic interactions, even with efficient binding.^32^ Here, the well-dispersed PVA particle network requires minimal recovery time to fully reconstruct itself following the cessation of shear.

**Figure 4:**
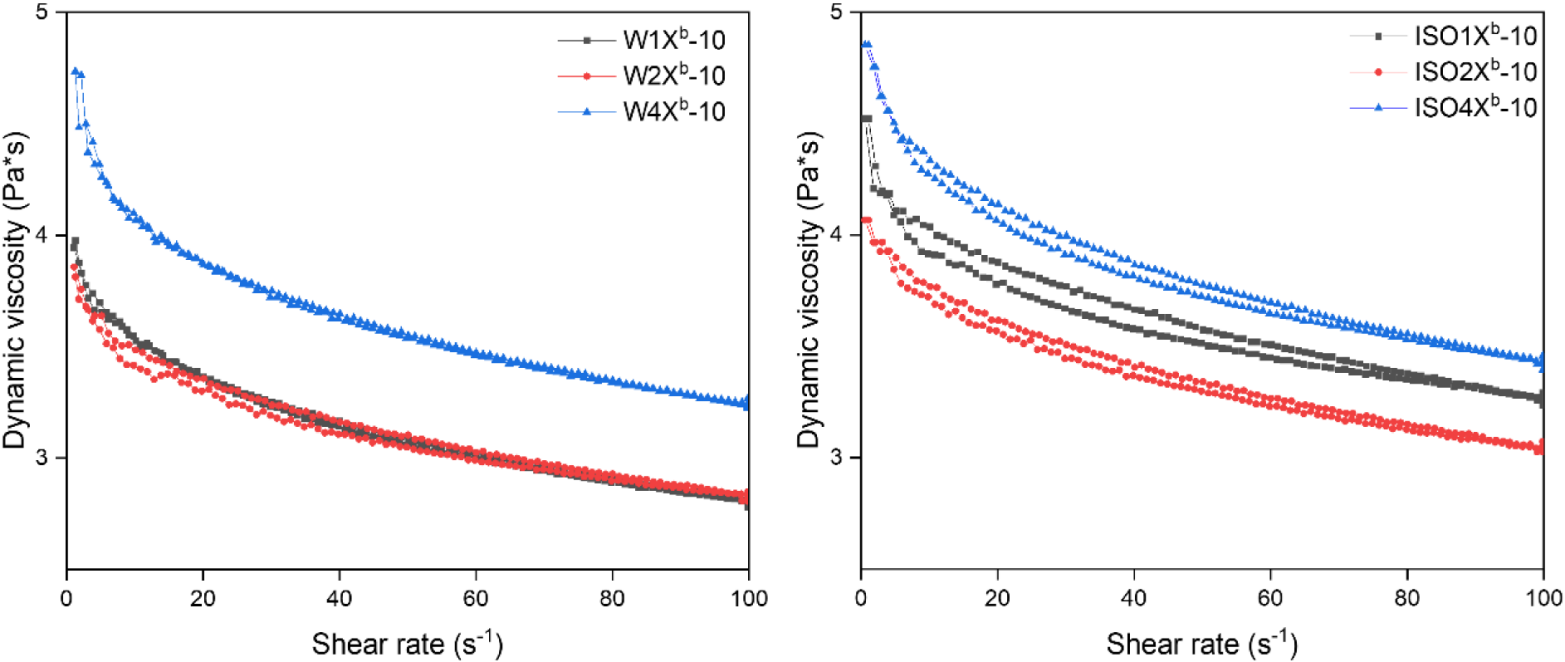
Rheological profiles of W1X^b^-10, W2X^b^-10, W4X^b^-10 (left) and ISO1X^b^-10, ISO2X^b^-10, ISO4X^b^-10 (right) ceramic-polymer suspension series at 25 °C. The curves demonstrate non-Newtonian pseudoplastic flow with increased viscosity at higher solids loading.

This validates the finding that a specific solid concentration exists where the high-molecular-weight PVA binder achieves optimal steric stabilization and interparticle friction, leading to the lowest viscosity. Conversely, the high viscosity at the maximum load is attributed to the dominant effect of the high solid volume fraction (packing fraction), which severely restricts fluid movement, overcoming any stabilization benefits. All samples exhibit a thixotropic behavior, where the return curve of the viscosity loop consistently tracks below the forward (upsweep) curve. This hysteresis indicates that the structured network of alumina particles and PVA chains requires finite time to fully recover after the applied shear stress is removed.^33^

The marked increase in this behavior, compared with the purely aqueous system, is highly likely linked to the cosolvent effect of ISO. ISO, being a poorer solvent for PVA compared to water, may more easily induce PVA chain contraction, promoting the formation of larger yet mechanically weaker flocculated structures through intermolecular association. These ISO-mediated structures are easily broken down (shear-thinned) during the upsweep, but their complex reconstruction kinetics cause delayed recovery during the downsweep, resulting in a wider hysteresis loop. The fact that the loop is open for all concentrations suggests that ISO has fundamentally changed the internal structure of the binder/particle network.^31^

This finding is critical for electrospinning, as the pronounced thixotropy means that the viscosity during the capillary flow will be significantly lower than the zero-shear viscosity, which is beneficial for stable flow through the nozzle. However, subsequent fiber stability will depend on the rapid structural recovery time of the PVA/alumina network to withstand electrohydrodynamic forces after exiting the nozzle. The sample with the smallest loop area (likely 2X loading) represents the most stable and easily recovered structure, offering the best compromise between low processing viscosity and structural integrity for nanofiber formation.

From the rheological behavior of the polymer suspensions (W1X^b^-10, W2X^b^-10, W4X^b^-10 and ISO1X^b^-10, ISO2X^b^-10, ISO4X^b^-10), their suitability for electrospinning was evaluated using the Ostwald-de Waele Power Law model analysis. The values presented in Table 3 confirm pseudoplastic (shear-thinning) behavior (*n* < 1), which is suitable for electrospinning for all samples, and the variations of this parameter were classified, reaching almost Newtonian behavior.

**Table 3:** Values from Ceramic-Polymer Suspensions Using the Ostwald-de Waele Power-Law Model Analysis^a^

sample ID	γ̇ at 1 s^–1^ (K) [Pa·s]	γ̇ at 10 s^–1^ [Pa·s]	calculated *n*	rheological class
W1X^b^-10	3.97	3.50	0.945	pseudoplastic
W2X^b^-10	3.85	3.48	0.956	near-Newtonian
W4X^b^-10	4.73	4.00	0.927	pseudoplastic
ISO1X^b^-10	4.50	4.00	0.949	pseudoplastic
ISO2X^b^-10	4.00	3.75	0.972	near-Newtonian
ISO4X^b^-10	4.85	4.33	0.951	pseudoplastic

a Consistency index (*K*) is numerically equivalent to the viscosity at a shear rate of 1 s^–1^ (Pa·s), *γ̇* is the viscosity at 10 s^–1^, and *N* (flow behavior index) is a dimensionless parameter indicating the degree of pseudoplasticity.

Comparative viscoelastic response of polymer suspensions in different solvent systems is presented in Figure 5. The W series demonstrates an enhanced elastic network with *G*’ values up to 30 Pa, while the ISO series displays a more stable and well-defined linear viscoelastic regime. The absence of a crossover point (*G*’ = *G*’’) within the measured range confirms that the solutions maintain their fluid-like state, a critical factor for consistent throughput for electrospinning processing. In all evaluated samples, the Loss Modulus (*G*’’) is consistently higher than the Storage Modulus (*G*’). This indicates that the suspensions behave as viscoelastic liquids across the measured frequency/stress range. For electrospinning, this dominance of the viscous component is typical for polymer solutions that must flow through a capillary and undergo extreme stretching. However, the presence of a measurable *G*’ confirms that there is a degree of internal structural network (polymer entanglement) necessary to stabilize the jet during the electrospinning process.

**Figure 5:**
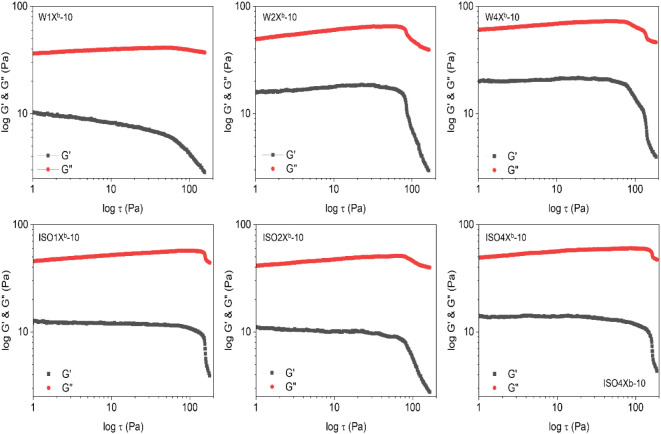
Amplitude sweep profiles show the Storage Modulus (*G*’) and Loss Modulus (*G*’’) as a function of shear stress (τ) for the W and ISO suspension series. The dominance of *G*’over *G*” across the Linear Viscoelastic Region (LVER) characterizes all samples as viscoelastic liquids.

On the other hand, the W series demonstrates viscoelastic character, as can be observed in Table 4, where the comparative results show that the *G*’ values (17–30 Pa) are significantly higher than those of the ISO series (9–13 Pa), indicating a more robust polymer entanglement network. Specifically, W1X^b^-10 exhibits the lowest loss tangent (tan *δ* = 1.57), which is advantageous for resisting capillary breakup during high-speed jet whipping. In contrast, the ISO series displays a sharper, more predictable transition from linear viscoelasticity to structural breakdown. This enhanced microstructural homogeneity ensures greater process reliability during the critical transition from the static Taylor cone to the dynamic spinning jet.

**Table 4:** Dynamic Moduli for Ceramic-Polymer Suspensions^a^

sample ID	storage modulus (*G*′) [Pa]	loss modulus (*G*″) [Pa]	loss tangent (tan δ = *G*″/*G*′)	rheological character
W1X^b^-10	30	47	1.57	viscoelastic liquid
W2X^b^-10	17	65	3.82	viscoelastic liquid
W4X^b^-10	21	72	3.43	viscoelastic liquid
ISO1X^b^-10	12	58	4.83	viscoelastic liquid
ISO2X^b^-10	9	51	5.67	viscoelastic liquid
ISO4X^b^-10	13	60	4.61	viscoelastic liquid

a These values represent the material’s response within the linear viscoelastic region (LVER) from Figure 5. Values of loss tangent >1 denotes a predominantly viscous (liquid-like) behavior.

The surface tension (γ) of the prepared suspensions was measured to evaluate its impact on the initiation and stability of the electrospinning jet, and the results are summarized in Table 5. All measured values, ranging from 58.3 mN/m to 64.2 mN/m, were significantly lower than that of pure water (74.8 mN/m), confirming the effective role of both the PVA binder and the incorporated surfactant in reducing the cohesive forces at the liquid–air interface. Notably, the initial solvent used during the ball-milling stage (W or ISO) showed minimal influence on the final γ of the electrospinning suspension, suggesting that γ is primarily dictated by the concentration of the high-molecular-weight polymer and the final aqueous medium. Crucially, a clear decreasing trend in γ was observed as the solids content was increased from 1X to 4X. This reduction is attributed to the higher concentration of surfactant effectively lowering the interfacial energy. In the context of electrospinning, lower γ facilitates the deformation of the droplet into the Taylor cone and reduces the required onset voltage. However, this advantage must be balanced against solution viscosity, as an excessively low γ can promote jet instability and the formation of defects like beads.

**Table 5:** Surface Tension (γ) and Conductivity (κ) from the Measured W and ISO Series Ceramic-Polymer Suspensions

sample ID	γ (mN·m^–1^)	κ (μS·cm^–1^)
W1X^b^-10	64.2 ± 0.9	1825 ± 2.5
W2X^b^-10	60.5 ± 2.0	1666 ± 6.0
W4X^b^-10	59.4 ± 2.4	1761 ± 7.6
ISO1X^b^-10	62.1 ± 1.4	1665 ± 1.5
ISO2X^b^-10	59.0 ± 0.8	892 ± 1.5
ISO4X^b^-10	58.3 ± 2.0	908 ± 2.6

The strong correlation between solids loading and electrical conductivity (κ) is an important factor that impacts the behavior of these complex suspensions within electrospinning processing. As the α-Al_2_O_3_ concentration increases, the conductivity rises markedly, a phenomenon primarily attributed to two factors: the increased concentration of the surfactant (K15 or K30) and the release of ionic species from the commercial alumina powder surface. Since the surfactant is dosed at a constant (1 wt %) relative to the alumina, higher solids loading introduces a greater density of amphiphilic molecules that, upon ionization in the aqueous PVA medium, significantly enhance the charge carrier density. Additionally, the high surface area of the ultrafine α-Al_2_O_3_ particles facilitates the desorption of residual ions or surface hydroxyl groups into the bulk solution. This elevated conductivity is beneficial for electrospinning as it increases the net charge of the jet, allowing for greater electrostatic repulsion forces that overcome the liquid’s surface tension more efficiently, leading to enhanced fiber thinning during the flight toward the collector.

## 3 Discussion

The transition from lab-scale feasibility to robust ceramic fiber production requires systematic optimization of suspension chemistry. The strategy reported in this work shows a series of steps for the improvement of the α-Al_2_O_3_ aqueous suspension’s suitability to form fibers. Reasonable feedback was established using SEM analysis for the morphology and average diameter determination. Therefore, the efforts were focused on the improvement of the solid’s contents.

The elaborated suspensions for electrospinning of solid oxide α-Al_2_O_3_ were systematically modified in order to improve the resulting ceramic fibers’ morphology, starting from a reasonable interval of concentrations based on previous reports.^19,34^ The first comparative experiment outcomes evaluating the solvent for the suspension (water or isopropanol) allow us to establish that using isopropanol enhanced the fiber morphology. Overall, according to the obtained results, the increase of the suspension’s loading with α-Al_2_O_3_ produces improvement and retention of the continuous fibrillar structure.

The suspensions, after the electrospinning process, were optimized by increasing the α-Al_2_O_3_ solids content (2X and 4X from the starting loading). For reaching these loading amounts and ensuring the stability of the α-Al_2_O_3_ particles, it was considered necessary to increase the molecular weight of the surfactant from K15 to K30. This step allows obtaining a single fiber-based structure, suggesting that the increase in the surfactant́’s molecular weight (10 000 to 58 000) causes different transition temperatures, avoiding the fusion of fibers.

Observed improvements in the fibrillar structure monitored by SEM allowed to establish the impact on average diameter. Similar values among the different samples were observed (Table 2) before the thermal treatment. However, the fiber morphology composed of polymer and α-Al_2_O_3_ is affected by the heating, causing melting and fusion of the fibers, thereby increasing the Ø. When the PVA concentration increases (from 8 to 10%wt), this effect is decreased but not eliminated. Micrometric fibers with improved fiber uniformity were achieved in the ISO2X^b^-10 and ISO4X^b^-10 sample conditions, based on the standard deviation values (25 and 27%, respectively). Even though the values of standard deviation can be considered high, it is worth establishing that the agglomeration of particles can promote rugosity along the fibers, which influences the average diameter, and broader distributions can be expected.

The rheological analysis reveals that all suspensions are highly suitable for electrospinning, as their consistency index (*K*) values range from 3.85 to 4.85 Pa·s (Table 3), within the industrial range of 1.0 to 10.0 Pa·s required for stable jet formation. However, the ISO series consistently exhibits higher *K* values than the W series, suggesting that ISO as a solvent enhances polymer chain entanglement, which typically results in more robust fibers with fewer beads.^35^

Regarding the flow behavior index (*n*), values ranging from 0.927 to 0.972 indicate that these suspensions are pseudoplastic (shear-thinning), though they lean toward Newtonian.^36^ The *n* values found in this work, particularly in ISO2X^b^-10 (*n* = 0.972), suggest a very stable Taylor cone that is less sensitive to pressure fluctuations. W4X^b^-10 stands out as the most processable variant for achieving finer diameters, as it possesses the lowest *n* value (0.927), allowing the fluid to thin out most effectively under high-voltage tension.

However, it is likely to produce fibers with higher structural integrity due to the stronger elastic component. However, the less-defined LVE might indicate a more complex internal structure that could lead to variations during long spinning sessions. The high tan δ values (4.6–5.7), combined with a clearly defined LVE, show viscoelastic behavior that is highly desirable in the electrospinning suspensions. The suspensions will flow easily (low resistance to deformation) while maintaining a very consistent and predictable microstructure, which is ideal for high-throughput industrial applications where uniformity is critical.^37^

According to the presented results, the molecular weight of PVP shows an influence on the formation of single electrospun α-Al_2_O_3_ fibers. PVP K-15 providesexcellent short-range stabilization and prevents the rapid clumping of particles, leading to a stable and highly dispersed suspension. This is highly desirable for processability in different additive manufacturing techniques, but it might not be ideal for the final ceramic fiber structure when using aqueous electrospinning processing. The physical properties of the suspensions were optimized, and the results favor stable jet formation. Surface tension values (γ = 58.3–64.2 mN/m) are ideal for aqueous-based spinning; they are low enough to allow the electric field to overcome cohesive forces and form a Taylor cone, yet high enough to maintain jet integrity. While the electrical conductivity (κ = 892–1825 μS/cm) sits at the high end of the industrial spectrum. This elevated conductivity increases the surface charge density, which significantly enhances the whipping instability. This phenomenon is essential for the mechanical stretching of the jet, allowing for the production of ceramic fibers with controlled diameters from commercially available powders.^38^

The lack of control and robustness observed at low loading (15–60%) values, combined with the low molecular weight, allows to establish that the electrospun α-Al_2_O_3_ fibers with single fiber morphology are promising at high solid content values in the initial suspensions, using aqueous binder PVA through electrospinning processing. In the case of PVP K30, this surfactant can create a different kind of interaction. The longer chains can bridge between multiple particles, creating a bridging flocculation effect and the formation of flocs.^39–44^ The state of the flocs in the solution is critical toward single fiber formation; the interconnection of flocs leads to a network of interconnected particles, according to the rheology characterization results. The right conditions for soft agglomeration and forming a continuous network were achieved in the ISO2X^b^-10 and ISO4X^b^-10 samples, preserving the single fiber morphology through the sintering process after the PVA is removed.

To the best of our knowledge, there are limited reports on the direct electrospinning of aqueous suspensions of commercial α-Al_2_O_3_ powders in the absence of sol–gel precursors. Therefore, it is relevant to report the experimental exploration of the implicated parameters for the adequate obtention of electrospun ceramic fibers. The data set obtained in this work can be consulted by the scientific and industrial community in order to design a new class of materials based on α-Al_2_O_3_ fibers, taking into account the presented concentration intervals for the suspension elaboration and the electrospinning parameters employed. The advantage of using commercially available materials with reduced environmental effects is based on the reduction of steps and costs for the advanced ceramic materials elaboration.

This strategy was intended for the fabrication of continuous fibers. However, the obtained materials are interesting from the point of view of their possible implementation as porous scaffolds for multifunctional applications. The obtained SEM images show an interesting interconnected α-Al_2_O_3_-based structure, forming intricated channels for fluid́s flow. The possibility of surface functionalization on porous scaffolds composed by chemically and thermally stable α-Al_2_O_3_ is highly desirable for robust and reusable devices for a wide range of technological applications.

## 4 Conclusions

The feasibility of direct aqueous α-Al_2_O_3_ electrospinning for the elaboration of matrices composed of single-fiber structures was confirmed using commercially available powders with PVA as a binder. The presented results demonstrate that it is achievable and controllable under the optimized experimental conditions established in this work.

Controlling particle agglomeration is a crucial but complex aspect of creating ceramic fibers from commercially available α-Al_2_O_3_ powders, especially through electrospinning. Achieving the right balance among the suspension properties and electrospinning parameters is key to prevent large clumps, while also ensuring enough controlled interaction to form a continuous network that can survive the sintering process.

The molecular weight of the PVP surfactant plays a decisive role in achieving a single-fiber morphology. Low-molecular-weight PVP K-15 yields a highly stable dispersion, which is generally not ideal, as the particles are too far apart to sinter effectively. Conversely, higher-molecular-weight PVP K-30 promotes bridging flocculation, establishing a necessary interconnected particle network (metastable state) within the polymer that preserves the fiber integrity during the sintering process.

This methodology establishes a valuable, environmentally conscious, and cost-effective data set for the scientific and industrial community, as it utilizes readily available commercial powders and avoids complex synthesis steps. The emergence of intermediate aluminum oxycarbide phases provides a promising avenue for functional material design. Rather than representing incomplete oxidation, these phases suggest that the residual carbon from the polymer/surfactant system can be used as a structural modifier. This in situ phase engineering opens the door for new applications where traditional alumina falls short, such as in high-temperature catalyst supports, nonwetting metallurgical filters, and reinforced ceramic matrix composites. Future studies should focus on controlling the oxycarbide-to-oxide ratio to tune the mechanical and electrical properties of these fibers for high-temperature filtration, catalysis supports, aerospace defense, matrix composites, and biomedical scaffolds, accomplishing specific industrial demands.

## 5 Methods

### 5.1 Materials

Aluminum oxide powder alpha-phase (α-Al_2_O_3_), 99.9% (metals basis), D_50_: 0.5–0.7 μm (typically); polyvinyl pyrrolidone (PVP) K15 (Mw ≈ 10,000), and K30 (Mw ≈ 58,000) as surfactants; isopropanol (99.5%) or distilled water as solvents; and poly­(vinyl alcohol) (PVA, Mw ≈ 146,000–186,000) as a binder were all chemicals purchased from Thermo Scientific Chemicals and used as received.

### 5.2 Solid Oxide-Based Suspensions Elaboration

Suspensions of α-Al_2_O_3_ were elaborated by milling for 4 h with different amounts of alumina powder and surfactant (PVP K15) to produce a 15 wt % solids load, using water and isopropanol as solvents. For increasing the α-Al_2_O_3_ content, the solids were increased 2X and 4X while maintaining the solvent content. Suspensions of α-Al_2_O_3_ 32 wt % using PVP K30 and isopropanol were elaborated as the starting concentration, and then the powder was increased 2X and 4X from the starting load.

PVA 10% and 8 wt % were prepared by dissolving PVA in hot water (80 °C) and stirring until complete dissolution (2 h). Then, a certain amount of Al_2_O_3_ suspension was combined with the PVA solutions and mixed with a planetary mixer (THINKY) at 2000 rpm for 5 min. Well-dispersed aqueous α-Al_2_O_3_-based suspensions were obtained for electrospun fiber fabrication.

### 5.3 Setting of the Electrospinning Processing Conditions

The electrospinning setup is composed of a Harvard 33 DDS syringe pump for controlled injection of the suspension through a metallic tip connected to the positive pole of a high-voltage power supply (0–30 kV) Matsusada EQ-30P1. The negative pole was connected to a rotating drum as a collector, isolated in a chamber at 25 °C and 60% relative humidity. The as-obtained PVA-α-Al_2_O_3_ fibers were calcined at 1500 °C for organic removal and ceramic sintering.

The designed methodology for the obtention of continuous α-Al_2_O_3_ fibers developed in this work is schematized in Figure 6. The flow rate (FR) and electric potential (kV) at 15 cm of working distance (WD) were varied until Taylor’s cone formation and adjusted for the evaluation of the suspension’s effect on the obtained fibers. The solids content, surfactant molecular weight, solvent, and PVA concentration were systematically varied for suspension properties, adjusting for α-Al_2_O_3_ obtention after the calcination, monitored using electron microscopy.

**Figure 6:**
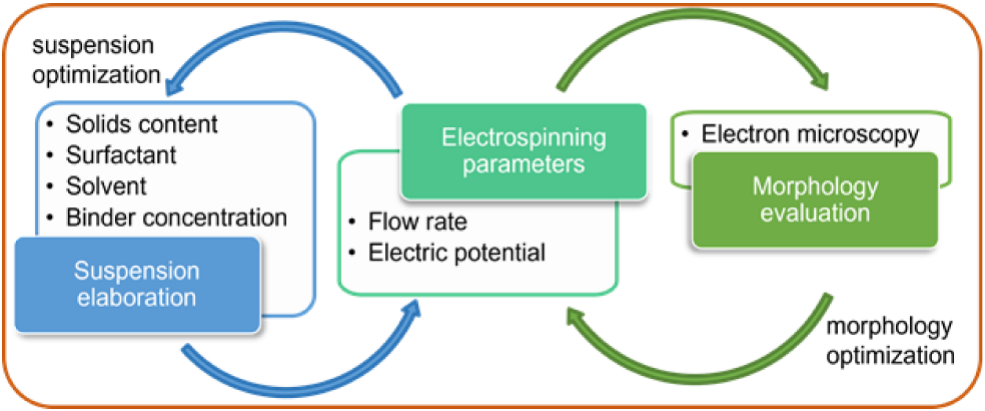
A schematic representation of the designed methodology to optimize the electrospinning parameters for the elaboration of ceramic fibers using commercially available alumina powders in aqueous suspensions.

### 5.4 Characterization

#### 5.4.1 Morphology

Scanning Electron Microscopy (SEM) was employed for the morphology monitoring of the differently processed samples. Fibers after and before calcination were observed in a field emission FEI InspectS50 electron microscope. Samples of the electrospun fibers after and before calcination were trimmed and fixed with carbon tape on a metallic holder. For adequate secondary electron images obtention, palladium coating was evaporated onto the surface. SEM images were processed using ImageJ software (1.54 g version) for average diameter determination. Statistical analysis of the average diameter (Ø) and standard deviation (s) was performed using Equations 1 and 2, respectively.

1 $$Ø=\frac{\sum d}{n}$$

2 $$s=\sqrt{\frac{\sum {\left(d-Ø\right)}^{2}}{n-1}}$$

where *d* is the measured diameter from SEM images, and *n* is the sample size (*n* = 200).

#### 5.4.2 Composition and Structure

Elemental composition was performed in a Quanta FEG 250 (ESEM) in low-vacuum mode, without any covering to avoid the presence of metallic covering in the data. The samples were fixed on Cu tape, and the observations were performed at 20 kV. FTIR was performed in an FTIR Bruker Vertex 70. XRD patterns were obtained in an X-ray diffractometer PANalytical Empyrean.

#### 5.4.3 Rheology

Rheological measurements were performed on a HAAKE MARS 40 rheometer with a parallel plate measuring system at 25 °C and 1.6 Hz. Measurements of apparent viscosity at 1 s^–1^ and 10 s^–1^ were employed to describe the non-Newtonian behavior of the samples using the Ostwald-de Waele Power-Law model (η = K·*γ̇* ^*n*‑1^) where *K* (consistency index) is numerically equivalent to the viscosity at a shear rate of 1 s^–1^ (Pa·s), *γ̇* is the viscosity at 10 s^–1^, and *n* (Flow Behavior Index) is a dimensionless parameter indicating the degree of pseudoplasticity (shear-thinning behavior). The index *n* was derived by linearizing the power-law equation (logη = log *K* + (*n* – 1)log *γ̇*).^45^

Storage Modulus (*G*’) and the Loss Modulus (*G*’’) as a function of shear stress (τ). The Linear Viscoelastic Region (LVER) was identified as the stress range where both moduli remained independent of the applied deformation. To evaluate the relative dominance of the viscous versus elastic components, the Loss Tangent (tan *δ*) was calculated as follows: $\tan\delta =\frac{G^{\prime \prime }}{G^{\prime }}$ , where values >1 denote a predominantly viscous (liquid-like) behavior.^46^

#### 5.4.4 Surface Tension (γ) and Conductivity (κ)

The surface tension was measured using the DSA25 Drop Shape Analyzer (KRÜSS, Germany), and the conductivity was measured using an EC-Meter GLP 31 (CRISON, Spain).

## Data Availability

Data used is available throughout the manuscript text.
